# Insight into the Phenolic Composition of Cabernet Sauvignon Grapevine Berries During Fermentation—Towards the Application of Winery By-Products for Antibacterial Purposes

**DOI:** 10.3390/antibiotics14030236

**Published:** 2025-02-25

**Authors:** Okba Hatem, Anita Seres-Steinbach, György Schneider, Éva Szabó, László Kőrösi

**Affiliations:** 1 Doctoral School of Health Sciences, Faculty of Health Sciences, University of Pécs, H-7622 Pécs, Hungary; 2 Department of Biochemistry and Medical Chemistry, Medical School, University of Pécs, H-7624 Pécs, Hungary; 3 Department of Medical Microbiology and Immunology, Medical School, University of Pécs, Szigeti Street 12, H-7624 Pécs, Hungary; 4 Research Institute for Viticulture and Oenology, University of Pécs, H-7634 Pécs, Hungary

**Keywords:** phenolic compounds, wine fermentation, wine by-product, grapevine seed, grapevine skin, Cabernet Sauvignon, antibacterial activity, *Listeria monocytogenes*, *Staphylococcus aureus*, high-performance liquid chromatography

## Abstract

Background: Wine production generates significant amounts of grape marc, which can serve as a potential source of bioactive compounds, including polyphenols. Objectives: In this study, we aimed to investigate the polyphenol content of skin and seeds separated from grape marc, and test their extracts against two significant bacteria, *Listeria monocytogenes* (*LM*) and *Staphylococcus aureus* (*SA*). Methods: A comprehensive analysis of the phenolic composition in the skin, seeds, and juice/wine derived from Cabernet Sauvignon grape berries was conducted over an 18-day fermentation period. High-performance liquid chromatography was performed to identify and quantify the main flavan-3-ols, flavonols, anthocyanins, and stilbenes. In addition, the total phenolic content (TPC) was determined by the Folin–Ciocalteu method. Results: The TPC of both seeds and skins significantly decreased over time. In parallel, the TPC in the wine gradually increased, indicating a release of phenolic compounds into the wine. We found that the TPC in seeds was consistently higher than in the skin at all examined time points. The main flavonoids in seeds were flavan-3-ols (catechin and epicatechin), while anthocyanins (delphinidin-, cyanidin-, petunidin-, peonidin-, and malvidin-3-*O*-glucoside) were the predominant ones in skins. Crude seed and skin extracts enriched in phenolics were prepared, of which only the crude seed extract was proven effective against *LM* and *SA*. Following the time-kill assay, our findings revealed that the minimal bactericidal concentration of the crude seed extract against *LM* was 5.02 mg/mL after 12 h incubation, demonstrating the eradication of the living bacterial cell number by ~6 log. A 24 h exposure time was required for complete inactivation of *SA*, but a lower concentration was sufficient (2.54 mg/mL). Conclusions: Grape waste remains a valuable source of polyphenols, with grape seeds, in particular, exhibiting significant antimicrobial activity against certain foodborne pathogens.

## 1. Introduction

Winemaking generates considerable amounts of nutrient-rich grape pomace—a by-product of stems, skins, and seeds—which is packed with bioactive phenolic compounds that hold immense potential for food and pharmaceutical applications [1]. Phenolics are characterized by a six-carbon aromatic ring attached directly to one or more hydroxyl (-OH) group. Except for simple phenolics, most of these compounds are found as polyphenols (PPs), containing more than one phenol unit [2]. In general, grape phenolic compounds can be divided into two major groups: flavonoids and non-flavonoids [3]. However, the composition of phenolic compounds in grapes, and consequently grape pomace, changes depending on grape variety and genetics, harvest conditions, and ripeness level [4]. These compounds play an important role in plant reproduction and protection against environmental changes, pathogens, and predators [5]. Additionally, dietary PPs exhibit antioxidant efficacy through scavenging free radicals, suppression of enzymes that are involved in free radical generation, activation of antioxidant enzymes, and chelating metal ions that induce oxidation reactions [6]. Grape seeds contain 8–20% of grape seed oil, which is a phenolics-rich edible oil with several applications in the cosmetic and pharmaceutical sector [7]. Indeed, the positive effects of grape seed extracts, such as anti-diabetic [8], anti-platelet [9], and anti-proliferative [10] effects have been attributed to their high PPs content. Furthermore, research revealed that PPs show several antibacterial mechanisms such as cell wall disruption, lipid membrane damage, or ion channels interference [11], making them appealing for use as naturally based antimicrobials.

Notably, there has been a growing demand for naturally derived food ingredients recently due to consumers’ awareness of synthetic food additives and their detrimental health effects [12]. Additionally, the increasing demand for fresh, minimally processed foods and instant meals raises much concern in the food industry to meet the food safety and food quality criteria [13,14]. In this context, microorganisms present a major challenge, as maintaining high standards of foodstuffs might be affected by the existence of microbes, including bacteria, yeasts, and molds, leading to changes in the physiochemical features of the product and food spoilage, which are associated with the deterioration of sensorial characteristics and nutritional values of foods [15]. In more serious cases, food contaminated with pathogens causes foodborne diseases (FBDs), which represent not only personal issues to the patient but also an added economic burden to public health due to the medication and hospitalization costs. Data from the World Health Organization (WHO) revealed that almost 1 in 10 people fall sick as a result of contaminated food annually, with more than 420 thousand death cases [16]. *Listeria monocytogenes* and *Staphylococcus aureus* are among the most common Gram-positive bacteria responsible for FBDs [17].

*Listeria monocytogenes* (*LM*) is one of the main pathogenic bacteria that cause acute FBD, posing a significant risk particularly to the elderly, pregnant women, newborns, and individuals with compromised immune systems. Although infection with *LM*, known as listeriosis, is rare, the mortality rate is relatively high (20–30%), even with antibiotic administration. Moreover, almost all patients infected with *LM* require hospitalization and monitoring in intensive treatment units [18]. *LM* is considered a resilient bacterium that can withstand harsh environments, such as low pH, high salt concentrations, and low temperatures [19].

*Staphylococcus aureus* (*SA*) causes FBD due to its ability to produce staphylococcal enterotoxins, which lead to food intoxication following ingestion, toxic-shock syndrome, or staphylococcal scaled skin syndrome based on the infection route [20]. These bacteria colonize the nose, throat, hair, and skin of around 50% of healthy asymptomatic individuals; thus, the main route of food contamination comes from food handlers [21]. The Centers for Disease Control (CDC) reported an annual incidence of 240,000 cases of *SA* infection in the USA, with 1000 cases requiring hospitalization and six deaths [22]. Even though it is a self-limiting condition, children and the elderly are prone to fatal dehydration and electrolyte imbalances [23]. Although exposing food to high temperatures kills *SA*, the enterotoxins are thermostable, and even a small intake (less than 200 ng) may trigger illness [24]. Therefore, maintaining good manufacturing practices and eliminating this bacterium from food is essential to prevent foodborne disease occurrence.

In this sense, antimicrobial preservatives represent an ideal solution to tackle these issues, as the addition of these agents prevent the growth of pathogens and increase the shelf life of the product, specifically when no cooking methods are applied [25]. Although the use of synthetic preservatives is under regulation, their total safety represents an ongoing debate topic [26]. This prompted a shift in research focus towards exploring naturally occurring compounds that may demonstrate antimicrobial efficacy. Additionally, food waste garnered attention as a vast source for extracting natural compounds, with an added advantage of decreasing the environmental burden of this waste [27].

Therefore, the primary aim of this research was to report the phenolic compound changes in grape pomace (seeds and skins), and grape juice (or wine) generated during the fermentation of grapes. We conducted an analysis of the total phenolic content (TPC) of the grape parts obtained from the vinification of by-products in Hungary during several fermentation points. Additionally, HPLC profiling of several individual PPs was performed in parallel, including flavan-3-ols, flavonols, anthocyanins, and stilbenes. Monitoring the dynamics of the TPC and/or individual compound alterations intended to control the fermentation effect on the by-product. *Vitis vinifera* cv. Cabernet Sauvignon was selected for this study, recognizing it as one of the most cultivated varieties around the world, which accounted for 5% of the total world area under vines, according to the International Organisation of Vine and Wine statistics in 2017 [28]. Moreover, our secondary aim was to evaluate the antimicrobial efficacy of the grape seeds and skins after applying a simple extraction procedure that generated a phenolic-rich crude seed and skin extract. These extracts were tested against the abovementioned microbes *LM* and *SA*. A kinetic study was then performed to determine the minimal bactericidal concentration (MBC) and the onset time of the effective crude extract.

## 2. Results

### 2.1. Total Phenolic Content (TPC)

#### 2.1.1. TPC of Seeds

**Figure 1:**
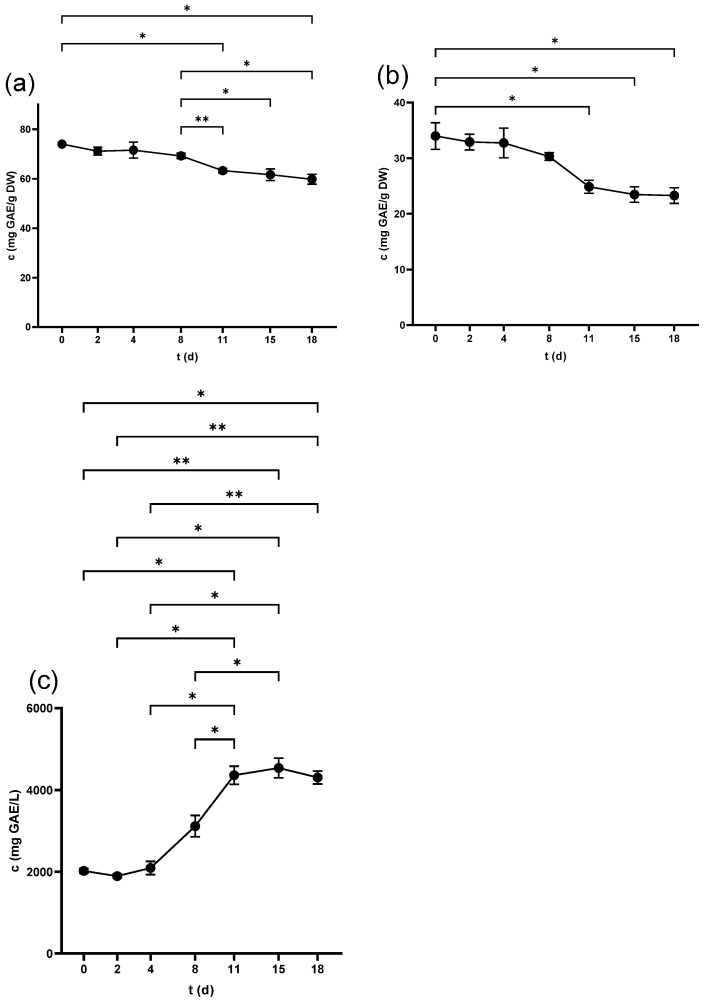
Total phenolic content (TPC) of (**a**) grape seed, (**b**) grape skin, and (**c**) grape juice throughout the fermentation period. * *p* < 0.05, ** *p* < 0.01.

The TPC of seeds scored the highest value in the non-crushed samples (87.5 ± 0.7 mg GAE/g DW), which decreased significantly after the crushing of the berries (74.0 ± 0.3 mg GAE/g DW). This figure witnessed a continuous fall to (59.8 ± 2 mg GAE/g DW) at 18 d as shown in Figure 1a. At the end of fermentation, the pomace was pressed, and the seeds’ TPC further decreased to 54.3 ± 2.2 mg GAE/g DW.

#### 2.1.2. TPC of Skins

Skin samples showed lower levels of TPC than seeds at all examined periods, where the highest values were recorded in non-crushed (44.7 ± 2.9 mg GAE/g DW) and freshly crushed samples (34.0 ± 2.4 mg GAE/g DW). A consistent decrease over time was observed (Figure 1b), reaching 23.3 ± 1.4 mg GAE/g DW by the end. Pressing led to a further significant reduction in the skin’s TPC, finishing at 19.6 ± 0.7 mg GAE/g DW.

#### 2.1.3. TPC of Juice

The TPC of juice started at the lowest value in the freshly squeezed juice (1307.5 ± 41.2 mg GAE/L) and increased significantly after 3 h (2024 ± 55.5 mg GAE/L). The TPC continued to increase in the following days as demonstrated in Figure 1c, peaking at 15 d (4541.1 ± 241.1 mg GAE/L), with no significant change at 18 d before pressing. However, the produced wine after pressing exhibited a marked increase in the TPC at 4875.5 ± 92.3 mg GAE/L.

### 2.2. Flavan-3-ols

#### 2.2.1. Flavan-3-ols in Seeds

**Figure 2:**
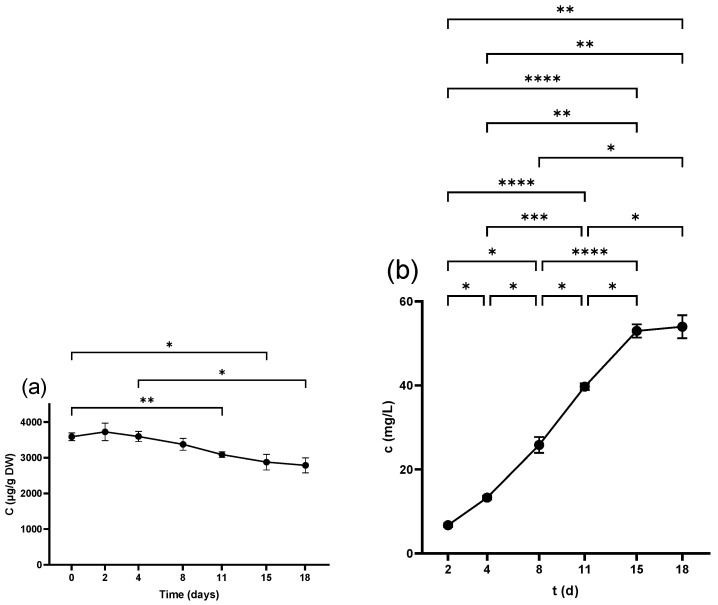
Catechin concentration in (**a**) grape seeds and (**b**) grape juice throughout the fermentation period. * *p* < 0.05, ** *p* < 0.01, *** *p* < 0.001, **** *p* < 0.0001.

**Figure 3:**
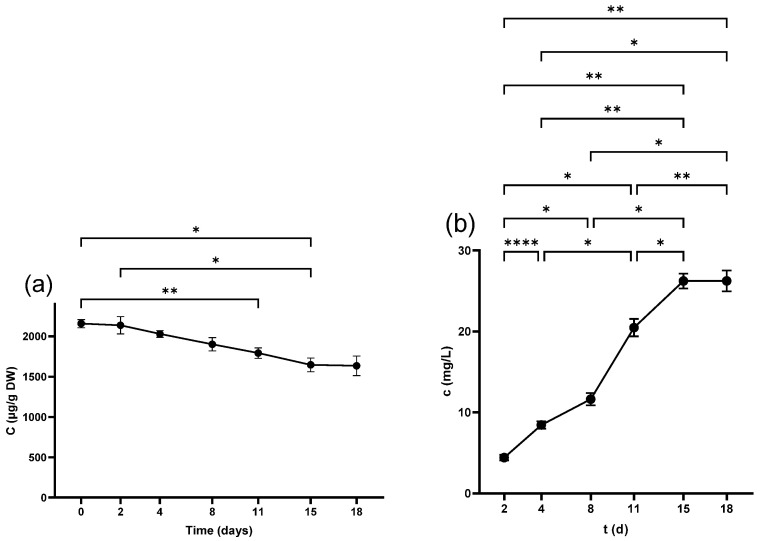
Epicatechin concentration in (**a**) grape seeds and (**b**) grape juice throughout the fermentation period. * *p* < 0.05, ** *p* < 0.01, **** *p* < 0.0001.

Catechin (Cat) and Epicatechin (Epi) were analyzed by HPLC and recorded the highest content in the non-crushed seeds at 3999.9 ± 147.9 µg/g DW and 2454.7 ± 100.5 µg/g DW, respectively. The level of both compounds decreased over time, with a statistically considerable decline from 11 d as seen in Figure 2a and Figure 3a. Table 1 illustrates that crushing and pressing resulted in a considerable reduction in Cat and Epi concentrations.

#### 2.2.2. Flavan-3-ols in Skins

Cat and Epi levels in the skins were under the limit of detection (LOD), with no effect of crushing or pressing, as shown in Table 2.

#### 2.2.3. Flavan-3-ols in Juice

Neither Cat nor Epi was detected in juice derived from freshly squeezed berries. Although Cat was measurable 3 h after crushing with 1.09 ± 0.1 mg/L, Epi reached a detectable level from 2 d, scoring (4.4 ± 0.4 mg/L). The concentration of both compounds displayed ongoing growth throughout the fermentation period (Figure 2b and Figure 3b) and pressing led to a further increase in the wine as detailed in Table 3.

### 2.3. Flavonols

#### 2.3.1. Flavonols in Seeds

HPLC analysis included nine main compounds in the flavonols group, namely myricetin-3-*O*-galactoside (M-gal), myricetin-3-*O*-glucoside (M-glc), quercetin-3-*O*-galactoside (Q-gal), quercetin-3-*O*-glucoside (Q-glc), quercetin-3-*O*-glucuronide (Q-glr), kaempferol-3-*O*-glucoside (K-glc), kaempferol-3-*O*-rutinoside (K-rut), kaempferol-3-*O*-glucuronide (K-glr), and isorhamnetin-3-*O*-glucoside (I-glc).

**Figure 4:**
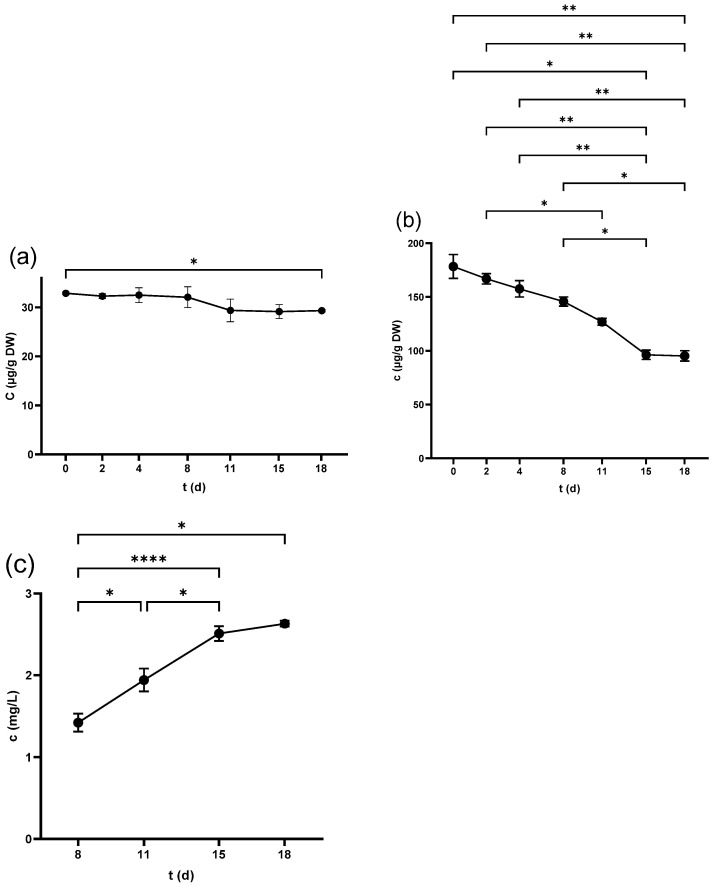
Quercetin-3-*O*-glucoside concentration in (**a**) grape seed, (**b**) grape skin, and (**c**) grape juice throughout the fermentation period. * *p* < 0.05, ** *p* < 0.01, **** *p* < 0.0001.

Q-glc was the only detectable compound in the seeds from the beginning, scoring (34.9 ± 0.9 µg/g DW) in the non-crushed samples. Figure 4a shows that the compound experienced an overall fall during fermentation. Crushing and pressing led to a substantial reduction in Q-glc as seen in Table 1, which also shows that all of the other flavonols were either undetectable or below the limit of quantification (LOQ) and only measurable at trace levels.

#### 2.3.2. Flavonols in Skins

**Figure 5:**
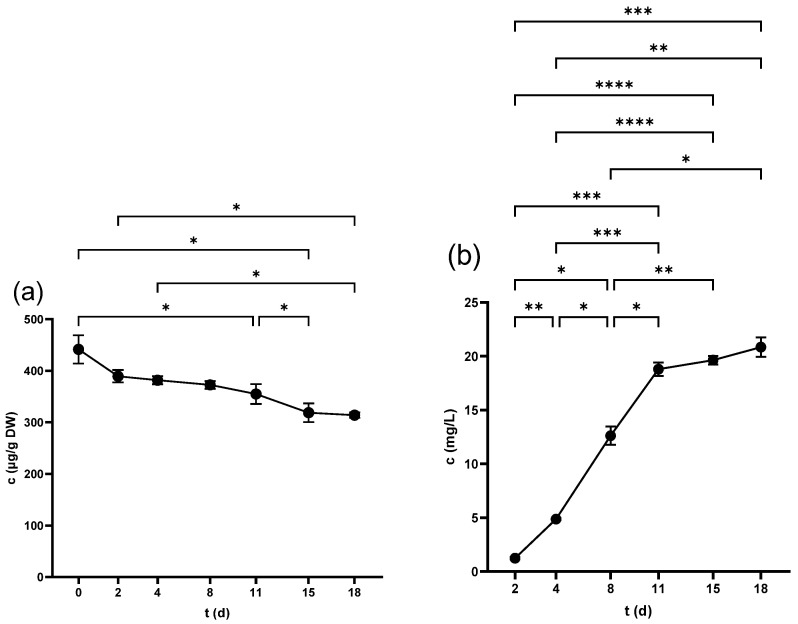
Myricetin-3-*O*-galactoside concentration in (**a**) grape skin and (**b**) grape juice throughout the fermentation period. * *p* < 0.05, ** *p* < 0.01, *** *p* < 0.001, **** *p* < 0.0001.

The major components in the non-crushed skins were M-gal, M-glc, Q-glc, and Q-glr, which experienced a considerable fall due to the crushing process. During winemaking, the content of these compounds continued to decrease (Figure 4b, Figure 5b, Figures S1a and S2a). Moreover, the content of these compounds decreased after pressing, as demonstrated in Table 2.

Q-gal and K-glc were only measurable in the non-crushed samples, and samples at 0 d, while they were detected in trace levels in the following days. Similarly, K-glr, K-rut, and I-glc were detected in trace concentrations from the beginning of the analysis. Detailed numbers are presented in Table 2.

#### 2.3.3. Flavonols in Juice

M-gal and M-glc were detected in the freshly squeezed juice and samples on day 0. M-gal was measurable from day 2, while M-glc from 4 d (Figure 5b and Figure S1b), and exhibited a continuous upward trend in the following days. Regarding quercetin derivatives, Q-glc and Q-glr were undetectable until 4 d. Their content increased from 8 d until the end of the fermentation as shown in Figure 4c and Figure S2a. The pressing process increased the concentration of all of these four compounds, while all other compounds were either undetectable or below LOQ during the whole process, as illustrated in Table 3.

### 2.4. Anthocyanins

#### 2.4.1. Anthocyanins in Seeds

Five major anthocyanins were quantified by HPLC, including malvidin-3-*O*-glucoside (Mvd-glc), delphinidin-3-*O*-glucoside (Dph-glc), cyanidin-3-*O*-glucoside (Cyd-glc), petunidin-3-*O*-glucoside (Ptd-glc), and peonidin-3-*O*-glucoside (Pnd-glc).

**Figure 6:**
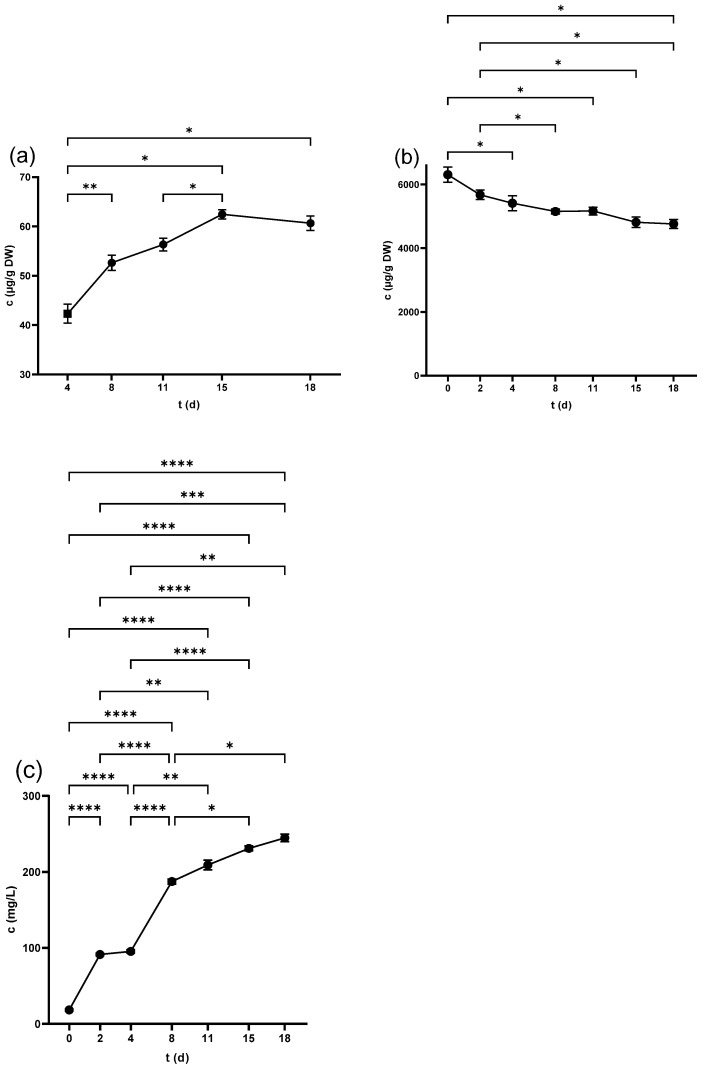
Malvidin-3-*O*-glucoside concentration in (**a**) grape seed, (**b**) grape skin, and (**c**) grape juice throughout the fermentation period. * *p* < 0.05, ** *p* < 0.01, *** *p* < 0.001, **** *p* < 0.0001.

Non-crushed seeds contained Mvd-glc solely in trace levels, while other anthocyanins were below LOD. Additionally, Mvd-glc rose to a noticeable level at 4 d, persisted in rising during fermentation (Figure 6a), and after pressing as demonstrated in Table 1, which also shows that the remaining compounds were either undetectable or measurable at trace levels.

#### 2.4.2. Anthocyanins in Skins

The anthocyanins were one of the predominant components of PPs in skins, with the highest value for Mvd-glc in the non-crushed samples (9135.4 ± 560.4 µg/g DW), followed by Dph-glc (3168.8 ± 204.3 µg/g DW), Ptd-glc (2152.3 ± 112.4 µg/g DW), Pnd-glc (932.1 ± 38.6 µg/g DW), and Cyd-glc (613.58 ± 22.2 µg/g DW). All of the compounds maintained a pattern of decreasing during fermentation as seen in Figure 6b and Figure S3 and dropped after pressing while remaining in the same order with a range 4598.2 ± 159.9 µg/g DW for Mvd-glc, to 283.1 ± 10.1 µg/g DW for Cyd-glc.

#### 2.4.3. Anthocyanins in Juice

Only Mvd-glc and Dph-glc were measurable in the freshly squeezed juice, while Cyd-glc, Ptd-glc, and Pnd-glc were quantifiable after 3 h of fermentation at 0 d. Crushing contributed to higher levels of Mvd-glc and Dph-glc. Although anthocyanins persisted in increasing during winemaking (Figure 6c and Figure S4), pressing increased only Mvd-glc concentration, while the other ones remained relatively unchanged. Detailed numbers are demonstrated in Table 3.

### 2.5. Stilbenes

#### 2.5.1. Stilbenes in Seeds

HPLC analysis involved two major stilbenes: resveratrol (Res) and piceid (Pic).

Neither of these compounds was detected in the seeds.

#### 2.5.2. Stilbenes in Skins

**Figure 7:**
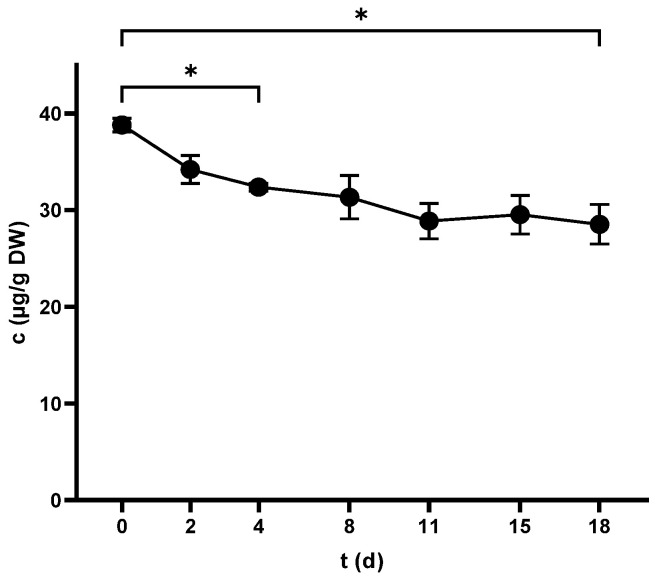
Piceid concentration in grape skins throughout the fermentation period. * *p* < 0.05.

Pic was the only measurable compound in the non-crushed samples at 74.9 ± 2.4 µg/g DW, while Res was detected at trace levels. Pic witnessed a decreasing trend during vinification (Figure 7), whereas Res was below LOD. Although crushing lowered the content of Pic, pressing had no significant effect (Table 2), and the compound finished at 26.6 ± 0.3 µg/g DW.

#### 2.5.3. Stilbenes in Juice

Pic was undetectable in the freshly squeezed juice but accumulated to a trace level at 18 days, while Res remained undetectable in all juice and wine samples (Table 3).

### 2.6. Enrichment of Phenolics in the Seed and Skin Extracts

The composition of the skin, seed, and juice samples has been described above. To prepare high concentrations of phenolic solutions for antimicrobial tests, crude extracts were prepared using seed and skin extracts as described in the Materials and Method section. Table 4 and Table 5 illustrate the HPLC results of the main phenolics in crude seed extract and crude skin extract, respectively. For the crude seed extract, the TPC was ten times greater than the TPC in non-crushed seeds at a level of 980.6 ± 15.0 mg GAE/g DW. Moreover, Cat and Epi increased by approximately 8- and 6-fold, respectively, compared to the native non-crushed seeds. In terms of flavonols, Q-glc had the highest content, followed by M-gal. Furthermore, Mvd-glc and Dph-glc contents tripled compared to samples after pressing, while the other anthocyanins remained under LOD. Finally, analyzing the stilbenes showed that resveratrol was not detected, while piceid scored trace levels.

The TPC of the crude skin extract was measured at 184.9 ± 11.3 mg GAE/g DW, which is a ninefold increase compared to the TPC in the skin after pressing. Additionally, Mvd-glc increased almost three times, while other anthocyanins remained at the same level, except for Dph-glc, which scored approximately half of the value in the final skin by-product.

### 2.7. Antimicrobial Experiments

We tested the antibacterial activity of the seeds and skin extracts prepared from the samples by simple extraction. Samples from three different time points (0 d, 8 d, and 18 d) were tested against two foodborne pathogens, *LM* and *SA*, after 24 h incubation. No significant antibacterial efficacy was recorded. Consequently, crude seed and skin extracts were prepared and applied to ensure a higher TPC and individual PPs content, as shown in Table 6 and Table 7, respectively

**Figure 8:**
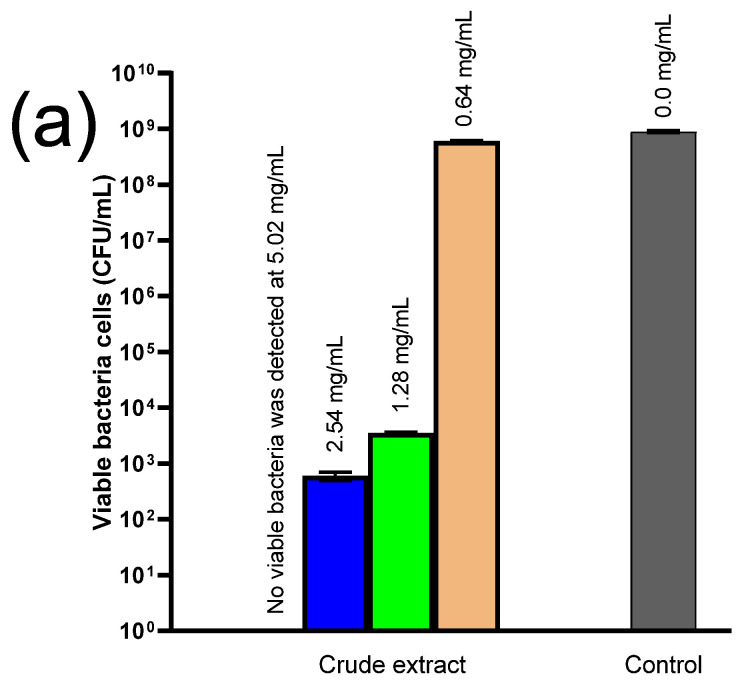

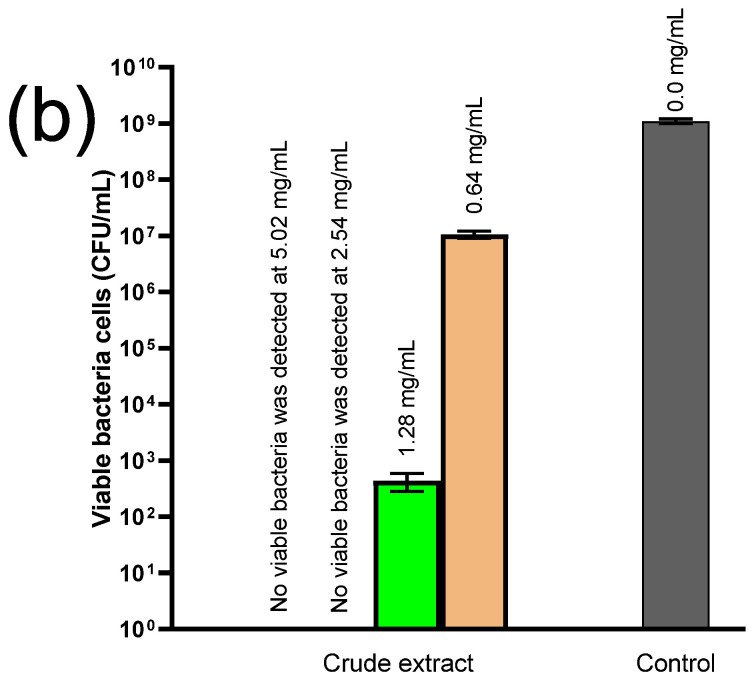
Antibacterial activity of crude seed extract against (**a**) *Listeria monocytogenes* (*LM*) and (**b**) *Staphylococcus aureus* (*SA*) after 24 h exposure. The numbers above the columns indicate the concentration of the crude extract.

Microbes were incubated for 24 h in the presence of the crude extracts, and viable bacterial cells were then enumerated. The crude skin extract was completely ineffective against both bacteria, whereas crude seed extract (Figure 8) at 5.02 mg/mL eradicated *LM* after 24 h incubation, while lower concentrations (2.54 and 1.28 mg/mL) decreased the bacterial number by almost 7 and 6 log units, respectively. In contrast, the lowest concentration (0.64 mg/mL) was completely ineffective, compared to the control.

Regarding *SA*, a crude seed extract content of 2.54 mg/mL was enough to completely kill the bacteria, which decreased by 7 log-folds when exposed to 1.28 mg/mL and 2 log-folds when exposed to 0.64 mg/mL of the crude seed extract after 24 h.

**Figure 9:**
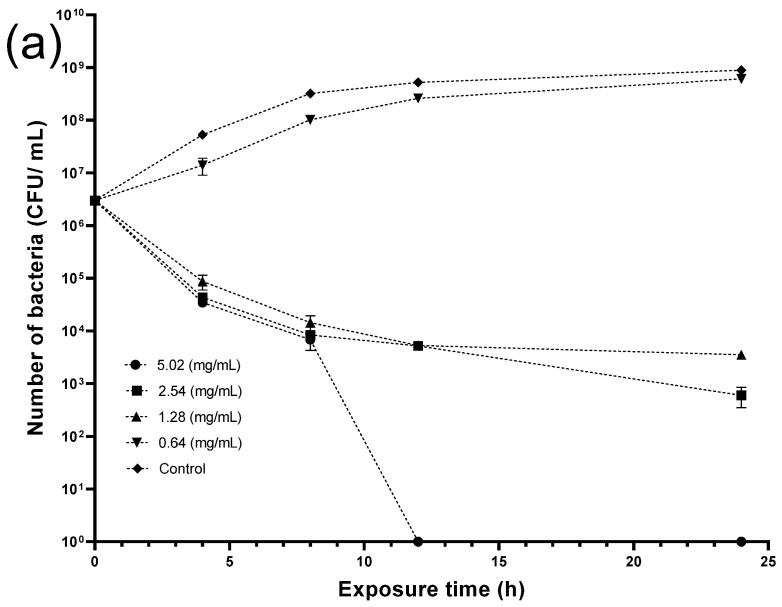

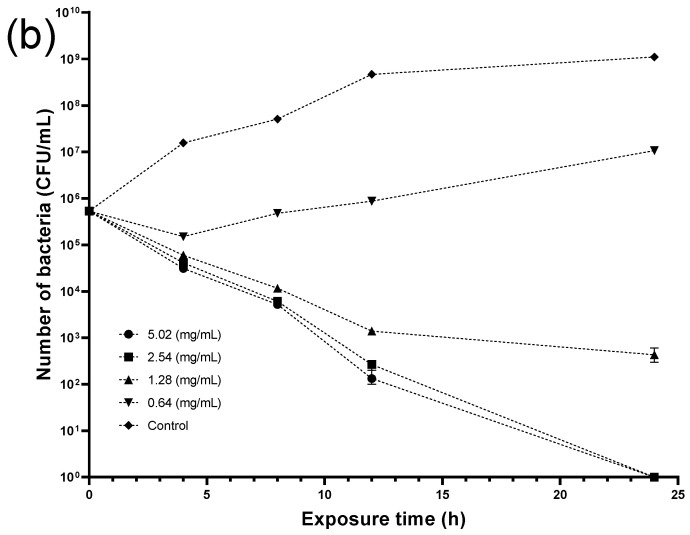
Time-kill curves of (**a**) *Listeria monocytogenes* (*LM*) and (**b**) *Staphylococcus aureus* (*SA*) treated with different concentrations of crude seed extract.

The following step included conducting a kinetic study to reveal the minimum bactericidal concentration (MBC) of the crude seed extract required to eradicate the bacteria at a certain time. Figure 9 shows that crude seed extract at 5.02 mg/mL could kill *Listeria* entirely after 12 h exposure. Moreover, crude extract at 2.54 and 1.28 mg/mL reduced the viable bacterial number from the fourth hour, whereas the lowest concentration (0.64 mg/mL) exhibited negligible activity, scoring comparable figures to the control throughout the experiment period. On the other hand, both concentrations (5.02 and 2.54 mg/mL) required a 24 h incubation time to eradicate *Staphylococcus aureus*. Although extract concentration at (1.28 and 0.64 mg/mL) reduced the bacterial cells at 4 h, the former concentration showed better antibacterial activity in the following hours, while the latter was weaker and significant bacterial growth was recorded.

## 3. Discussion

Vinification is one of the essential sectors in the food industry that generates nearly 20 million tons of wine by-products annually [29]. The environmental and economic awareness from one side, and human demand for naturally based ingredients from the other side, encourage the utilization of this waste, which is abundant in bioactive compounds, including phenolics. The composition of these compounds differs based on multiple conditions, such as climate and seasonal circumstances [30] grape variety [31], winemaking process, and fermentation method [32], as well as the analyzed grape part [33].

Based on the available literature, this research is the first to thoroughly document the dynamic changes in the TPC and individual PPs in seeds and skins of CS grapes, starting with the freshly harvested samples, and continuing the analysis during an 18 d fermentation process. We aimed to investigate the effect of fermentation on these compounds, and the possibility to utilize the by-products generated after winemaking, with a specific focus on antibacterial activity, as previous research showed that phenolic-rich grape pomace extracts demonstrate synergetic antibacterial mechanisms with antibiotics [34].

The compositional analysis revealed that the TPC of seeds and skins decreased by almost 30% and 50%, respectively, when comparing the fresh samples derived from non-crushed berries and the final by-products. However, these by-products are still considered as a valuable source for PPs, especially seeds, which demonstrated a higher TPC than skin at each time point. In accordance with our findings, it has been revealed that the concentration of phenolic compounds was higher in the seeds than in the skins of grape pomace for various varieties, including CS [35]. In addition, a study compared the TPC of wine grape pomace skins extracted by two methods, and revealed that the TPC of CS ranged from 26.7 ± 1.8 to 12.7 ± 0.5 mg GAE/g DM [36], which is in the same range of our study. As expected, the TPC of juice increased during fermentation, reaching (4875.5 ± 92.3 mg GAE/L) after pressing. All phenolic compounds increased in the juice over time due to their release from the seeds and skins. It is worth noting that the TPC might increase as a result of the production of new phenolics in the wine, during fermentation and aging, such as the reaction of Mvd-glc with pyruvic acid and acetaldehyde, leading to the synthesis of vitisin A and vitisin B, respectively [37].

Our analysis revealed that the main PPs in grape seeds were flavan-3-ols, i.e., Cat and Epi, which matches the findings of previously published data [38]. Catechins induce oxidative stress and DNA damage in bacteria, engaging several bacterial targets, such as cell membrane, bacterial enzymes, and virulence factors. For instance, they can cause bacterial cell membrane alteration through destroying the lipid membrane, leading to cell death [39]. Another bactericidal mechanism involves the competitive inhibition of dihydrofolate reductase, which is an essential enzyme that mediates the synthesis of the purines and consequently the bacterial DNA [40]. Additionally, bacterial toxins, which are considered an important virulence factor, can be suppressed by catechins either by a direct bind, or indirectly through blocking their release or enhancing toxins breakdown [41]. The concentration of Cat was almost (1.6–1.7) times higher than that of Epi in the non-crushed seeds, and this ratio remained during different fermentation times. Although Cat and Epi were reported by Mattive and colleagues to be the major components of seeds flavanols of red grape varieties, including CS, their portions were similar (49.6 for Cat and 49.3% for Epi). Moreover, minor concentrations of flavan-3-ols in the skins of the red wine grapes were detected, and the predominant one was Cat [42], while these compounds were under the limit of detection in our work, in line with the findings of Rockenbach and colleagues research, who could not detect Cat or Epi in the skin of CS pomace [35]. With regard to juice, our results showed that non-crushed samples did not contain catechin or epicatechin, while other researchers revealed that both of the compounds were present in the fresh juice of CS, at 2.146 ± 0. 215 μg/mL and 3.043 ± 0.30 μg/mL, respectively [43]. This can be explained by differences in sample preparation. It should be noted that our freshly squeezed juice samples were obtained from peeled and deseeded berries, preventing any damage to the seeds and subsequent release of catechins. In fact, it was proposed that concentrations of Cat and quercetin could be used as indicators of regional characteristics in CS wines. This suggestion was based on the analysis of wines from eight Balkan regions, which showed that phenolic content varied according to each region’s specific agroclimatic conditions and winemaking practices [44]. Flavan-3-ols displayed a consistent upward trend in juice during fermentation, finishing at (60.8 ± 1.5 mg/L) and (32.4 ± 1.3 mg/L) for Cat and Epi, respectively, which are in accordance with the reported levels of French wines [45].

On the other hand, the predominant PPs in skins were anthocyanins, which exhibit several antibacterial actions by interfering with bacterial metabolic pathways and disrupting bacterial cell wall, and their efficacy is strongly influenced by their specific chemical structure [46]. We revealed that the highest concentration among these compounds was for Mvd-glc, similar to earlier research that reported Mvd-glc as the most common anthocyanin in *V. vinifera* [32]. The subsequent anthocyanins in skin were Dph-glc, followed by Ptd-glc, Pnd-glc, and Cyd-glc. Comparable results of fresh skins of CS have been reported, with the following concentrations in the same order (19.15 ± 0.007, 5.04 ± 0.023, 3.23 ± 0.004, 2.46 ± 0.005, and 0.75 ± 0.016 expressed as malvidin-3-*O*-glucoside mg/g DW). The same sequence was recorded in the skins of CS pomace after winemaking, with Mvd-glc decreasing to 1.10 ± 0.008 mg/g DW, and trace levels for Cyd-glc [47]. All of these compounds were absent in the non-crushed seeds, with the exception of Mvd-glc at a trace level. This compound reached a quantifiable level at 4 d, and increased in the following days, peaking after pressing. Dph-glc was measurable only at 18 d. Although we detected these compounds in the seeds, another study indicated that anthocyanins were not detectable in the seeds of CS pomace [35]. We demonstrated that anthocyanins continued to increase in juice during fermentation, following the same arrangement in the final product, from Mvd-glc (293.3 ± 6.3 mg/L) as the highest, to Cyd-glc (4.5 ± 0.0 mg/L) as the lowest. A study analyzed CS red wine anthocyanin profile and reported that Mvd-glc (137.815 ± 0.396 mg/L) was the predominant compound. Nevertheless, the next anthocyanin compounds were Ptd-glc (5.047 ± 0.014 mg/L), Dph-glc (2.801 ± 0.011 mg/L), Pnd-glc (2.295 ± 0.001 mg/L), and Cyd-glc (0.814 ± 0.010 mg/L) [48].

We also observed considerable levels of flavonols in skins, which have been reported to demonstrate antibacterial efficacy against both Gram-positive and Gram-negative bacteria in a chemical structure-dependent manner [49]. Our findings showed four compounds that were quantifiable following descending order of concentration: M-gal, M-glc, Q-glc, and Q-glr, during the whole study, while Q-gal and K-glc were measurable only in the non-crushed and freshly crushed skins. The other compounds were recorded at trace levels. These findings are in accordance with reported data elsewhere [50]. Regarding flavonols in seeds, Q-glc was the only measurable one from the beginning, while other compounds were either undetectable or measurable at trace levels. Flavonols might play a role in the protection of the plant against UV [51], which explains the higher content of the skins comparing to the seeds. Moreover, M-gal and M-glc, the predominant flavonols in skins, reached measurable levels in juice at 2 d and 4 d, respectively, while Q-glc and Q-glr were above LOQ at 8 d. All of these figures witnessed a constant increase, finishing at a range between (23.6 ± 1.0 mg/L) for M-gal, and (3.8 ± 0.1 mg/L) for Q-glc. These finding are in partial agreement with a previous study which revealed that Q-glc, Q-glr, and M-glc were the major flavonols of CS berry skins and red wine [52].

Finally, since stilbenes are grapevine phytoalexins, i.e., produced as a defense mechanism in the plants against biotic and abiotic conditions [53], we measured these compounds due to their high potency to be utilized as antimicrobials. Neither Res nor Pic was present in the seeds. On the other hand, Pic was detected in non-crushed skins, and declined over fermentation, while Res was undetectable after crushing. Consequently, neither of the compounds was detectable in freshly squeezed juice samples, and while Pic accumulated to a trace level at 18 d, Res remained under LOD during this study. These findings are in correspondence with a previous work, where resveratrol was undetectable in either seeds or skins of CS pomace [35]. In fact, these compounds are predominant in other grape parts, such as canes, with antibacterial efficacy against *LM* [54].

We analyzed the efficacy of skin and seed samples from three time points against two food-borne pathogens: *LM* and *SA*. The efficacy was negligible; therefore, crude seed and skin extracts were prepared to increase PPs content. Interestingly, a crude skin extract with a final Mvd-glc of (63.40 ± 0.29 µg/mL) in the bacterial suspension showed no efficacy. HPLC analysis of the crude seed extract, which showed antibacterial effects against both microbes, revealed an approximate 18-fold rise in TPC compared to the content of after-pressing seeds. It has been documented that antibacterial efficacy and the TPC of the grape seed extract were correlated [55].

The main phenolic compound in the crude seed extract was Cat (32,025.3 ± 154.2 µg/g DW), followed by Epi (14,652.5 ± 42.8 µg/g DW). Additionally, two anthocyanins (Mvd-glc and Dpg-glc) and one flavonol compound (Q-glc) increased by (3–4) times in comparison to the seeds after the fermentation process. Interestingly, M-glc, which was at trace levels in the after-pressing seeds, scored considerable level (62.6 ± 0.4 µg/g DW) in the crude seed extract. The rationale behind this is that modification of the extraction technique leads to variation in phenolics yield [56]. For instance, we increased the mass of seeds and applied different solvents when producing the crude seed extract. In addition, given that M-glc is the predominant flavonol in skins, it may adsorb onto seeds during fermentation. Therefore, when we increased the mass of seeds in the extraction, M-glc concentration accumulated beyond LOQ.

When evaluating the efficacy of the crude seed extract against *LM*, a concentration of 5.02 mg/mL was demanded for eradicating the bacteria after 24 h incubation, whereas lower concentration (2.54 mg/mL) was enough to exhibit this action against *SA*. Requiring higher levels to kill *LM* have also been reported when examining the efficacy of Salix babylonica hydroalcoholic extract, where the MBC was 50 mg/mL and 100 mg/mL for *SA* and *LM*, respectively [57]. Additionally, the minimum inhibitory concentration (MIC) of ginseng extract was 90 mg/mL against *SA*, and 40 mg/mL against *LM* [58]. Moreover, a previous study found that commercially available grape seed extract powder, which showed significant efficacy against *SA*, was ineffective against *LM* [59], while other researchers found an inhibitory effect of grape seed extract against *SA* [55]. These conflicts emerge from a complex interplay of factors connected to the antimicrobial agent applied—which we have already discussed—and the characteristics of the microorganism under study. *LM* is a non-spore-forming, facultative anaerobe, Gram-positive rodlike bacterium. Based on the somatic and flagellar antigen structure, *LM* strains can be categorized into 13 serotypes, which can be further divided into four genetically different lineages, of which lineages I and II are involved in human disease. [60] *LM* is capable of infecting host cells due to several virulence factors, such as adhesion and invasion of cells through surface proteins known as internalins (In1A and In1B), escaping phagosomes and entering cytoplasm through extracellular listeriolysin O protein and phospholipases (PlcA and PlcB), and spreading from cell to cell through actin polymerization facilitated by ActA protein [61]. *SA* is a Gram-positive clamp-forming coagulase-positive cocci shaped bacterium, which is responsible for a variety of human diseases and can lead to life-threatening cases of bacteremia and pneumonia. [62]. However, *SA* foodborne disease is mainly associated with contamination by heat-stable enterotoxins [63]. Foodborne *SA* strains exhibited 27 virulence factors, of which the biggest portion is connected to phage lysis (autolysins and N-acetylmuramoyl-L-alanine amidases), adherence and invasive (phenol-soluble modulins), immune evasion (extracellular fibrinogen-binding proteins), and invasion (virulence factor EsxA) [64].

Our kinetic study showed that *LM* was eradicated after 12 h of incubation at 5.02 mg/mL (160.77 ± 0.77 µg/mL Cat, 73.56 ± 0.21 µg/mL Epi), whereas *SA* required 24 h of exposure, but a lower concentration (2.54 mg/mL, 81.34 ± 0.39 µg/mL Cat, 37.22 ± 0.10 µg/mL Epi) was enough. Unlike our research, the majority of studies lack reports of individual PPs contained in the extracts tested against microorganisms, with some exceptions, such as Kao et al., who demonstrated similar results after applying grape seed extract (with approximately 75–80% oligomeric proanthocyanidins and 3–5% monomeric proanthocyanidins) against *SA* [65].

A proposed mechanism of the grape seed extract against *LM* may involve interfering with the metabolic pathways of *LM*, such as increasing the lactic acid and putrescine, and decreasing leucine, isoleucine, glutamate, and acetoin [66]. On the other hand, it was hypothesized that grape seed extract demonstrates efficacy against *SA* through the partial inhibition of dihydrofolate reductase activity and interfering with the metabolic pathway of folate-mediated one-carbon, contributing to intracellular tetrahydrofolate reduction in *SA* [65].

Grapeseed extract is considered Generally Recognized as Safe (GRAS) by the FDA [67], and has been proposed to be used to enhance the bacterial inactivation as a synergetic factor. Previous studies revealed that Epi alone was not effective against *LM* or *SA*, while showing a non-significant inhibition when combined with streptomycin [68]. In our earlier publication, we demonstrated that ε-viniferin, a predominant stilbenic compound in grape canes, required five-times higher concentration to exhibit the same antibacterial effect of the crude cane extract [54]. On the other hand, combining both Cat and epicatechin-gallate improved the antibacterial activity of β-lactam antibiotics against Methicillin-resistant Staphylococcus aureus (MRSA) in vitro and in vivo [69]. Additionally, Zhao et al. found that treating *LM* in a liquid broth with a combination of nisin (2000 IU/mL) and 1% grape seed extract drastically lowered the bacterial concentration by 4.49 log CFU/mL within just 10 min. However, when nisin and grape seed extract were applied separately, they achieved only a minimal reduction in *LM* by 0.63–2.88 log CFU/mL [66]. In addition, a recent paper confirmed the synergetic antibacterial effect of using grape seed extract in combination with a non-thermal technology, cold atmospheric plasma, against *LM* wild type and other mutagenic strains [70]. Therefore, applying the whole extract instead of an isolated phenolic compound appears to be more effective in increasing the antibacterial efficacy of antibiotics and antibacterial technologies.

In this study, we demonstrated that grape by-products are vital sources packed with phenolics; the seeds hold the highest levels of flavan-3-ols, whereas the skins are loaded with anthocyanins. These residues are of a high potency to be utilized as natural bio-sources, specifically fermented grape seeds as natural antibiotics. We demonstrated the MBC and the onset time of bacterial reduction. Moreover, we provided an insight into the required time for eradication of two foodborne pathogens: *LM* and *SA*.

Extraction of grape seeds produced a catechins-rich crude extract, which eradicated *LM* and *SA* at around 160 µg/mL and 80 µg/mL of Cat concentration, respectively. In contrast, anthocyanins-rich crude skin extract at around 65 µg/mL of Mvd-glc showed no such effect. Grape seed by-products, therefore, hold significant potential for antimicrobial application in the food and pharmaceutical industries.

## 4. Materials and Methods

### 4.1. Chemicals and Reagents

Acetonitrile and methanol (Promochem Optigrade, LGC Standards GmbH, Wesel, Germany) were ≥99.9%, gradient grade, suitable for HPLC. Flavan-3-ols ((+)-catechin and (−)-epicatechin)), anthocyanins (delphinidin-3-*O*-glucoside, cyanidin-3-*O*-glucoside, petunidin-3-*O*-glucoside peonidin-3-*O*-glucoside, and malvidin-3-*O*-glucoside), stilbenes (resveratrol and piceid), and flavonols (myricetin-3-*O*-galactoside, myricetin-3-*O*-glucoside, quercetin-3-*O*-galactoside, quercetin-3-*O*-glucoside, quercetin-3-*O*-glucuronide, kaempferol-3-*O*-rutinoside, kaempferol-3-*O*-glucoside, isorhamnetin-3-*O*-glucoside, and kaempferol-3-*O*-glucuronide) with HPLC purity were purchased from Extrasynthese (Genay, France). Glacial acetic acid (≥99.9%) and ethanol were purchased from Molar Chemicals Ltd., Halásztelek, Hungary. Folin–Ciocalteu′s phenol reagent, sodium carbonate (≥99.5%) and gallic acid (≥98.0%), were purchased from Merck Life Science Kft., Budapest, Hungary. High-purity deionized water was obtained using a LaboStar 7 TWF-UV ultrapure water system (SGWasseraufbereitung und Regenerierstation GmbH, Barsbüttel, Germany).

### 4.2. Sample Preparation

*Vitis vinifera* cv. Cabernet Sauvignon berries were harvested at technological ripeness from the Villány region in Hungary. The harvesting was conducted manually, and the berries were then transferred to the winery where they underwent sorting to remove the leaves, damaged berries, and undesirable substances. The berries were then destemmed and crushed using a Zambelli EMME 60 destemmer-crusher machine (Zambelli Enotech s.r.l., Camisano Vicentino, Italy), and the resulting crushed berries were placed in open vats for the fermentation process. A 5% aqueous solution of potassium metabisulfite was administered gradually, resulting in a final concentration of 10 g/hL, followed by the addition of a Uvavital™ complex yeast nutrient (Danstar Ferment AG., Fredericia, Denmark) at 10 g/hL. An additional 10 g/hL of the yeast nutrient was added two days later. The natural wild yeasts were allowed to initiate the fermentation, but in the later phase (on the 6th day), we also applied Lalvin™ EC-1118 *Saccharomyces cerevisiae* (Lallemand Inc., Montréal, QC, Canada) at 20 g/hL. To ensure maximal flavor and color extraction during fermentation, the juice was pumped over the cap once each day. Samples were collected over an 18 d fermentation period of wine-making.

Samples included non-crushed berries and 3 h freshly crushed berries at 0 d, marc and juice obtained at several time points (2, 4, 8, 11, and 15 d), and marc and juice obtained at the end of fermentation before and after pressing at 18 d. For juice samples, non-crushed freshly squeezed samples represent samples squeezed and stored directly obtained from manually peeled and seeded berries, preventing any damage to the seeds or skins and the subsequent release of phenolics, while freshly crushed samples at 0 d represent samples collected after 3 h of the fermentation process. Three individual samples were collected at each time point. Seeds and skins were carefully separated from the pomace manually, while the juice was collected directly from the fermentation tanks. All samples were frozen in liquid nitrogen, then transported to the lab and stored at −80 °C until further processing. Seeds and skin samples were freeze-dried for 24 h using a ScanVac CoolSafe 110-4 Freeze Dryer (LaboGene ApS, Allerod, Denmark) and grinded using a simple coffee grinder. Freeze-dried samples were also stored at −80 °C. Must and wine samples were filtered through a 0.22 µm PES syringe filter (FilterBio^®^, Labex Ltd., Budapest, Hungary) before HPLC analysis.

### 4.3. Sample Extractions

Sample extraction was conducted based on the methods previously developed in our laboratory [54,71,72,73], with slight modifications. The extraction of flavan-3-ols, flavonols, and TPC involved adding 50 mg freeze-dried seed powder or 100 mg freeze-dried skin powder placed into 2 mL Eppendorf tubes and then 1.5 mL of a mixture of methanol/water/acetic acid (70/29.5/0.5, *v*/*v*/*v*%). The resulting suspension was sonicated using an Elma Transsonic T 460 ultrasonic bath (Singen/Hohentwiel, Germany) with a noise frequency of 35 kHz for 10 min, and then centrifuged at 20,660× *g* for 5 min. The supernatant was collected in a 5.0 mL volumetric flask. The procedure was repeated two more times, and the final volume was made up to 5.0 mL using the solvents mixture used for the extraction. This procedure was also applied to extract the anthocyanins from skin.

Due to the low content of anthocyanin in seeds, the sample-to-solvent ratio was increased. For anthocyanin extraction from seeds, (100 ± 1 mg) of the freeze-dried seed powder was mixed with 0.5 mL of a mixture of methanol/water/acetic acid (70/29.5/0.5, *v*/*v*/*v*%). The resulting suspensions were sonicated for 10 min and then centrifuged for 5 min. The procedure was repeated, and the supernatant was collected and made up to the calibration line of 1.0 mL volumetric flask using the same solvent mixture. For stilbenes extraction from seeds and skins, the same steps were followed, replacing the solvent with ethanol/water (50/50 *v*/*v*%).

### 4.4. Preparation of Seed and Skin Crude Extract and HPLC Analysis

This procedure was performed to increase the TPC in the extract. To illustrate, approximately 100 g of freeze-dried seed powder was mixed with 200 mL of a mixture of acetonitrile/water (70:30 *v*/*v*%). After sonication for 10 min and centrifugation for 5 min, the supernatant was collected and subsequently freeze-dried for 24 h to obtain a crude seed extract powder. Similarly, the crude skin extract was prepared with a minor modification in the volume of the solvents, as 400 mL was required to dissolve the freeze-dried skin powder.

### 4.5. Total Phenolic Content (TPC) Determination

A Folin–Ciocalteu assay [73] with minor modifications was carried out to determine the TPC of the grape seed and skin after extraction (Section 4.3), while juice or wine samples were used directly in the assay. To perform the modified Folin–Ciocalteu assay, a volume of 5 µL of the seed extract or 100 µL of the skin extract, or 10 µL of juice or wine was pipetted to 2.5 mL of distilled water, followed by the addition of 250 µL of Folin–Ciocalteu reagent, 1 mL of 20 *w*/*v*% sodium carbonate aqueous solution, and 1 mL of distilled water, with rigorous mixing after each step. The obtained solutions were placed in a dark place for 30 min, and then the absorbance was measured at 750 nm by using a UV-visible spectrophotometer (Shimadzu UV-1800). Gallic acid solutions in the concentration range 0.2–5 mg/L with five concentration levels were used for calibration. The TPC was expressed as milligrams of gallic acid equivalents per gram of weight of the freeze-dried powder (mg GAE/g). The same steps were followed to determine the TPC in crude extracts, after preparing appropriate dilutions.

### 4.6. HPLC Analysis of Individual PPs

HPLC analysis of individual phenolics was performed based on our previous work [54,71,72,73]. Instrumentation: HPLC analysis was performed on a Shimadzu Prominence UFLC system (Shimadzu Co., Kyoto, Japan) consisting of an on-line degassing unit (DGU-20A5R), pump (LC-20AD), column oven (CTO-20AC), autosampler (SIL-20AC HC), diode array detector (SPD-M20A), and fluorescence detector (RF-20A).

Calibration curves were obtained by measuring high-purity reference materials with known concentrations. Limits of detection (LOD) and quantification (LOQ) were determined according to Equations (1) and (2), respectively.

(1) $$LOD=\frac{3.3\sigma }{S}$$

(2) $$LOD=\frac{10\sigma }{S}$$

where *σ* is the standard deviation of response and *S* is the slope of the calibration curve. The LOD and LOQ values, together with the concentration ranges of the calibration, the corresponding equations of calibration curves, and correlation coefficients (R^2^) are presented in Table S1. Spiking of the extracts with reference materials for all matrices was performed to check the compound identity. The results were expressed in μg individual phenolic compound per mg dry weight (DW) of seed, skin, or its solid extracts. Phenolics in juice and wine samples were expressed in μg/mL (or mg/L).

#### 4.6.1. HPLC Analysis of Flavan-3-ols

The chromatographic separation of flavan-3-ols was conducted on a Kinetex^®^ 2.6 μm XB-C18 100 Å, 100 × 4.6 mm LC column (Phenomenex, Torrance, CA, USA). The column temperature was kept at 25 °C. Gradient elution was applied using 0.5% (*v*/*v*) acetic acid (A) and a mixture of acetonitrile (99.5% *v*/*v*) and acetic acid (0.5% *v*/*v*) (B). Gradient elution started at 100% (*v*/*v*) A, ramping up to 15% B over 15 min. The flow rate of the mobile phase was kept at 1.0 mL/min; the volume of the injected sample was 5 μL. For the fluorometric detection of catechin and epicatechin, the excitation and emission wavelengths were 278 nm and 360 nm, respectively.

#### 4.6.2. HPLC Analysis of Anthocyanins

The chromatographic separation was conducted on a Kinetex^®^ 2.6 µm Polar C18 100 Å, 100 × 4.6 mm LC Column (Phenomenex, Torrance, CA, USA) [71]. The column temperature was kept at 40 °C. Gradient elution was applied using 10% (*v*/*v*) formic acid (A) and a mixture of acetonitrile (90% *v*/*v*) and formic acid (10% *v*/*v*) (B). Gradient elution started at 97% (*v*/*v*) A, ramping up to 21% B over 37 min. The flow rate of the mobile phase was kept at 1.0 mL/min; the volume of the injected sample was 5 µL. For the evaluation of anthocyanins, the absorbance values acquired at 530 nm were used.

#### 4.6.3. HPLC Analysis of Stilbenes

The HPLC analysis of stilbenes was conducted in accordance with the methods outlined in [54]. The separation was conducted on a Kinetex^®^ 2.6 μm XB-C18 100 Å, 100 × 4.6 mm LC column (Phenomenex, Torrance, CA, USA). The column temperature was kept at 25 °C. Gradient elution was applied using 0.5% (*v*/*v*) acetic acid (A) and a mixture of acetonitrile (99.5% *v*/*v*) and acetic acid (0.5% *v*/*v*) (B). Gradient elution started at 100% (*v*/*v*) A, ramping up to 56% B over 20 min. The flow rate of the mobile phase was kept at 1.0 mL/min; the volume of the injected sample was 5 μL. Resveratrol and piceid were quantified using the absorbance values acquired at 306 nm.

#### 4.6.4. HPLC Analysis of Flavonols

The HPLC analysis of flavonols was conducted following the methods outlined in [73] with a slight modification. The separation was conducted on a Kinetex^®^ 2.6 μm XB-C18 100 Å, 100 × 4.6 mm LC column (Phenomenex, Torrance, CA, USA). The column temperature was kept at 25 °C. Both isocratic and linear gradient elution steps were applied using 0.25% (*v*/*v*) acetic acid (A) and a mixture of acetonitrile (99.75% *v*/*v*) and acetic acid (0.25% *v*/*v*) (B). Elution began at 100% (*v*/*v*) A for 3 min, then ramped up to 7% B over 8 min, followed by a further increase to 16% B over 2 min. Finally, an isocratic step was applied using 16% B for 18 min. The flow rate of the mobile phase was kept at 1.0 mL/min; the volume of the injected sample was 20 μL. Flavonols were quantified using the absorbance values acquired at 350 nm.

### 4.7. Antimicrobial Experiments

#### 4.7.1. Bacterial Strains and Cultural Conditions

For the experiments, two Gram-positive bacterium strains were used. The *Listeria monocytogenes* strain ATCC35152 is a widely used strain type, isolated from poultry in the United Kingdom and having the serotype 1/2b. The *Staphylococcus aureus* strain USA300 is an epidemiologically important human strain that became the predominant strain type of Methicillin-Resistant *Staphylococcus aureus* (MRSA) circulating in the United States by 2011 [74].

Overnight (12 h) blood-based (BB) agar cultures of *LM* and Luria–Bertani (LB) agar cultures of *SA* were used to set their optical densities to 0.2 at 600 nm. The numbers of living bacteria (CFU—colony forming unit) were determined from the obtained suspensions before each experiment. For that, 10-fold serial dilutions were made using phosphate-buffered saline (PBS) and 10 μL from each dilution step was dispensed onto BB and LB agar plates for *LM* and *SA*, respectively, and were incubated aerobically overnight at 37 °C. All the bacterium suspensions were incubated in a shaking incubator under aerobic conditions at 37 °C during the experiment. CFU determinations were also performed after each antibacterial test as described below. Each experiment was repeated three times.

#### 4.7.2. Antibacterial Testing and the Time-Kill Assay

This study examined the antibacterial efficacy of various grape seed and skin extracts according to the methods of our previous work [54]. First, the antibacterial efficacy of the non-crushed seeds and skins, seeds, and skins in the middle of the fermentation period (8 d), seeds and skins after pressing. The solutions were prepared by adding 100 mg of seeds or skin powder to 500 µL of 50% *v*/*v* ethanol. Additionally, two crude extracts were prepared by adding (200 mg) of the crude powder of seed or skin to 1 mL of 50% *v*/*v* ethanol. Before antibacterial experiments, the TPC was measured in the crude extract, and HPLC analysis was conducted to determine individual PPs as mentioned in (Section 4.4).

To examine the antibacterial efficacy, the following steps were taken for both microbes. Initially, 2 mL of LB medium and 20 µL of the bacterial suspensions were placed in sterile tubes, followed by pipetting a range of volumes (50, 25, 12.5, and 6.25 µL) from the previous stock solutions. For control, 50 µL of 50% *v*/*v* ethanol was used. The number of viable cells in the *LM* initial stock suspension was (3.1 × 10^8^ CFU/mL), while it was (9.2 × 10^7^ CFU/mL) for *SA*. The tested suspensions, on the other hand, scored (3 × 10^6^ CFU/mL) and (5.3 × 10^5^ CFU/mL) for *LM* and *SA*, respectively. The tubes were incubated for 24 h. Afterward, a sequence of dilution in PBS was prepared for each tube and 10 µL of each dilution was dispensed onto BB agar plates. The bacterial colonies were enumerated following a subsequent 24 h incubation.

Based on preliminary experiments, the crude seed extract solution with (200 mg/mL) concentration was chosen as the stock solution to conduct the time-kill assay, which was carried out for 24 h following the same steps and the same dilutions that resulted in the final concentrations shown in Table 6. Viable bacterial cells were counted in the bacterial suspensions incubated with the crude seed extract after 0, 4, 8, 12, and 24 h of exposure. MBC was defined as the concentration of extract needed to completely eliminate bacteria within a specified time frame.

### 4.8. Statistical Methods

Experiments were carried out at least in triplicates (n ≥ 3), and data are expressed as mean ± standard deviation. Statistical analysis was performed using SPSS 26.0 for Windows (SPSS Inc., Chicago, IL, USA). Multiple comparisons were conducted using One-Way ANOVA (Analysis of Variance) and Tukey’s HSD post hoc test. Asterisks illustrate significance as follows: * *p* < 0.05, ** *p* < 0.01, *** *p* < 0.001, **** *p* < 0.0001. *t*-test was applied to compare two groups at the level of significance *p* < 0.05.

## 5. Conclusions

Winemaking generates a considerable amount of grape by-products, and although the fermentation process reduces the polyphenol content in grape marc, the remaining seeds and skins still provide a significant source of these bioactive compounds, making them highly valuable for further utilization. Our research demonstrated that grape seeds consistently contained higher levels of phenolics compared to grape skins across all stages of processing. Notably, the distribution of polyphenols differed between the two parts, with flavan-3-ols being the predominant compounds in seeds, while anthocyanins were most abundant in skins. Furthermore, the crude seed extract at 5.02 mg/mL (160.77 ± 0.77 µg/mL Cat and 73.56 ± 0.21 µg/mL Epi) exhibited significant antibacterial activity against two food-borne pathogens, whereas the crude skin extract showed no such effect. These findings highlight the promising potential of grape by-products, particularly seeds, as a sustainable source of natural antibacterial agents, encouraging their utilization in developing functional and eco-friendly applications.

## Figures and Tables

**Table 1:** Concentrations of individual phenolic compounds in seed samples at different stages of berries processing. Samples were collected from non-crushed berries and after 3 h, and 18 d crushing of the berries with and without of application of pressing.

Phenolic Compound in Seed	Non-Crushed	Freshly Crushed (3 h)	Before Pressing (18 d)	After Pressing (18 d)
	(µg/g DW)			
Catechin	3999.9 ± 147.9 ^ab^	3589.0 ± 108.0 ^a^	2788.0 ± 210.3 ^c^	2081.4 ± 110.1 ^bc^
Epicatechin	2454.7 ± 100.5 ^ab^	2157.9 ± 50.6 ^a^	1635.5 ± 121.7 ^c^	1167.6 ± 71.1 ^bc^
Quercetin-3-*O*-galactoside	nd	nd	nd	nd
Quercetin-3-*O*-glucoside	34.9 ± 0.9 ^ab^	32.9 ± 0.3 ^a^	29.3 ± 0.3 ^c^	25.7 ± 0.5 ^bc^
Quercetin-3-*O*-glucuronide	nd	nd	trace	trace
Myricetin-3-*O*-galactoside	nd	trace	trace	trace
Myricetin-3-*O*-glucoside	nd	trace	trace	trace
Kaempferol-3-*O*-glucoside	nd	nd	nd	nd
Kaempferol-3-*O*-rutinoside	nd	nd	nd	nd
Kaempferol-3-*O*-glucuronide	nd	nd	nd	nd
Isorhamnetin-3-*O*-glucoside	nd	nd	nd	nd
Malvidin-3-*O*-glucoside	trace	trace	60.7 ± 1.5 ^a^	77.3 ± 1.1 ^a^
Delphinidin-3-*O*-glucoside	nd	trace	11.4 ± 0.4 ^a^	13.5 ± 0.2 ^a^
Cyanidin-3-*O*-glucoside	nd	nd	nd	nd
Petunidind-3-*O*-glucoside	nd	nd	trace	trace
Peonidin-3-*O*-glucoside	nd	nd	nd	nd
Resveratrol	nd	nd	nd	nd
Piceid	nd	nd	nd	nd

Values are expressed as mean ± std. The same superscript letters within a row indicate statistical significance (*t*-test, *p* < 0.05); trace: trace amount of the compound was detected; nd: not detected.

**Table 2:** Concentrations of individual phenolic compounds in skin samples at different stages of berries processing. Samples were collected from non-crushed berries and after 3 h, and 18 d crushing of the berries with and without of application of pressing.

Phenolic Compound in Skin	Non-Crushed	Freshly Crushed (3 h)	Before Pressing (18 d)	After Pressing (18 d)
	(µg/g DW)			
Catechin	nd	nd	nd	nd
Epicatechin	nd	nd	nd	nd
Quercetin-3-*O*-galactoside	571.3 ± 23.9 ^ab^	441.6 ± 27.4 ^a^	314 ± 5.39 ^c^	275.1 ± 6.4 ^bc^
Quercetin-3-*O*-glucoside	445.6 ± 19.5 ^ab^	388.5 ± 1.5 ^a^	213.2 ± 9.9 ^c^	190.5 ± 5.9 ^bc^
Quercetin-3-*O*-glucuronide	56.2 ± 11.8 ^a^	34.3 ± 5.1 ^a^	trace	trace
Myricetin-3-*O*-galactoside	228.6 ± 12.1 ^ab^	178.3 ± 11.1 ^a^	95.2 ± 4.9 ^c^	68.4 ± 3.0 ^bc^
Myricetin-3-*O*-glucoside	265.5 ± 10.1 ^ab^	233.7 ± 10.1 ^a^	121.7 ± 7.1 ^c^	101.6 ± 0.9 ^bc^
Kaempferol-3-*O*-glucoside	56.41 ± 10.03 ^a^	37.73 ± 9.12 ^a^	trace	trace
Kaempferol-3-*O*-rutinoside	trace	trace	trace	trace
Kaempferol-3-*O*-glucuronide	trace	trace	trace	trace
Isorhamnetin-3-*O*-glucoside	trace	trace	trace	trace
Malvidin-3-*O*-glucoside	9135.4 ± 560.4 ^ab^	6307.9 ± 238.4 ^a^	4761.4 ± 242.1 ^c^	4598.2 ± 159.9 ^bc^
Delphinidin-3-*O*-glucoside	3168.8 ± 204.3 ^ab^	2362.0 ± 153.1 ^a^	1724.7 ± 101.4 ^c^	1471.8 ± 86 ^bc^
Cyanidin-3-*O*-glucoside	613.6 ± 22.2 ^ab^	531.4 ± 15.1 ^a^	317 ± 9 ^c^	283.1 ± 10.1 ^bc^
Petunidind-3-*O*-glucoside	2152.3 ± 112.4 ^ab^	1505.4 ± 37.1 ^a^	1089.4 ± 81.3 ^c^	887.8 ± 11.6 ^bc^
Peonidin-3-*O*-glucoside	932.1 ± 38.6 ^ab^	773.6 ± 39.8 ^a^	392 ± 17.9 ^c^	331.6 ± 12.5 ^bc^
Resveratrol	trace	nd	nd	nd
Piceid	74.9 ± 2.4 ^ab^	38.80 ± 0.7 ^a^	28.5 ± 2.0	26.6 ± 0.3 ^b^

Values are expressed as mean ± std. The same superscript letters within a row indicate statistical significance (*t*-test, *p* < 0.05); trace: trace amount of the compound was detected; nd: not detected.

**Table 3:** Concentrations of individual phenolic compounds in juice samples at different stages of berries processing. Non-crushed sample derived from pressed flesh of manually peeled and deseeded berries. Samples were also collected after 3 h, and 18 d crushing of the berries with and without of application of pressing.

Phenolic Compound in Juice/Wine	Non-Crushed	Freshly Crushed (3 h)	Before Pressing (18 d)	After Pressing (18 d)
	(mg/L)			
Catechin	nd	1.9 ± 0.1	54 ± 2.7 ^a^	60.8 ± 1.5 ^a^
Epicatechin	nd	trace	26.3 ± 1.3 ^a^	32.4 ± 1.3 ^a^
Quercetin-3-*O*-galactoside	trace	trace	20.8 ± 0.9 ^a^	23.6 ± 1.0 ^a^
Quercetin-3-*O*-glucoside	trace	trace	9.7 ± 0.3 ^a^	11.1 ± 0.2 ^a^
Quercetin-3-*O*-glucuronide	nd	nd	trace	trace
Myricetin-3-*O*-galactoside	nd	nd	2.6 ± 0.0 ^a^	3.8 ± 0.1 ^a^
Myricetin-3-*O*-glucoside	nd	nd	5.0 ± 0.3 ^a^	5.7 ± 0.2 ^a^
Kaempferol-3-*O*-glucoside	nd	nd	trace	trace
Kaempferol-3-*O*-rutinoside	nd	nd	nd	nd
Kaempferol-3-*O*-glucuronide	nd	nd	nd	nd
Isorhamnetin-3-*O*-glucoside	nd	nd	nd	nd
Malvidin-3-*O*-glucoside	9.6 ± 0.3 ^ab^	18.4 ± 1.2 ^a^	244.8 ± 4.9 ^c^	293.3 ± 6.3 ^bc^
Delphinidin-3-*O*-glucoside	2.6 ± 0.2 ^ab^	4.7 ± 0.3 ^a^	45.4 ± 2.7	48.8 ± 4.3 ^b^
Cyanidin-3-*O*-glucoside	trace	1.4 ± 0.0	4.6 ± 0.1	4.5 ± 0.0
Petunidind-3-*O*-glucoside	trace	3.1 ± 0.2	42.2 ± 2.3 ^a^	43.7 ± 3.1 ^a^
Peonidin-3-*O*-glucoside	trace	3.0 ± 0.2	16.7 ± 0.2	17.2 ± 1.1
Resveratrol	nd	nd	nd	nd
Piceid	nd	nd	trace	trace

Values are expressed as mean ± std. The same superscript letters within a row indicate statistical significance (*t*-test, *p* < 0.05); trace: trace amount of the compound was detected; nd: not detected.

**Table 4:** Concentrations of the individual phenolics of crude seed extract.

Phenolic Compound	c (µg/g DW)
Catechin	32,025.3 ± 154.2
Epicatechin	14,652.5 ± 42.8
Quercetin-3-*O*-galactoside	trace
Quercetin-3-*O*-glucoside	89 ± 0.6
Quercetin-3-*O*-glucuronide	trace
Myricetin-3-*O*-galactoside	62.6 ± 0.4
Myricetin-3-*O*-glucoside	trace
Kaempferol-3-*O*-glucoside	nd
Kaempferol-3-*O*-rutinoside	nd
Kaempferol-3-*O*-glucuronide	nd
Isorhamnetin-3-*O*-glucoside	nd
Malvidin-3-*O*-glucoside	249.5 ± 1.8
Delphinidin-3-*O*-glucoside	39.9 + 0.1
Cyanidin-3-*O*-glucoside	nd
Petunidind-3-*O*-glucoside	trace
Peonidin-3-*O*-glucoside	trace
Resveratrol	nd
Piceid	trace

Values are expressed as mean ± std.; trace: trace amount of the compound was detected; nd: not detected.

**Table 5:** Concentrations of the individual phenolics of crude skin extract.

Phenolic Compound	c (µg/g DW)
Malvidin-3-*O*-glucoside	12,628.7 ± 58.2
Delphinidin-3-*O*-glucoside	776.3 ± 4.3
Cyanidin-3-*O*-glucoside	274.8 ± 2.5
Petunidind-3-*O*-glucoside	985.2 ± 8.4
Peonidin-3-*O*-glucoside	385.2 ± 7

Values are expressed as mean ± std.

**Table 6:** Calculated concentrations of total phenolic compounds and individual phenolics of crude seed extract in the bacterial suspensions.

Total and Individual Phenolics	Concentration of Crude Seed Extract in Antibacterial Test (mg/mL)			
	5.02	2.54	1.28	0.64
Total phenolic content (mg GAE/mL)	4.92 ± 0.08	2.49 ± 0.04	1.26 ± 0.02	0.63 ± 0.01
Catechin (µg/mL)	160.77 ± 0.77	81.34 ± 0.39	40.99 ± 0.18	20.50 ± 0.10
Epicatechin (µg/mL)	73.56 ± 0.21	37.22 ± 0.10	18.76 ± 0.05	9.38 ± 0.03
Quercetin-3-*O*-glucoside (µg/mL)	0.45 ± 0.00	0.23 ± 0.00	0.11 ± 0.00	0.06 ± 0.00
Myricetin-3-*O*-galactoside (µg/mL)	0.31 ± 0.00	0.16 ± 0.00	0.08 ± 0.00	0.04 ± 0.00
Malvidin-3-*O*-glucoside (µg/mL)	1.25 ± 0.01	0.63 ± 0.00	0.32 ± 0.00	0.16 ± 0.00
Delphinidin-3-*O*-glucoside (µg/mL)	0.20 ± 0.00	0.10 ± 0.00	0.05 ± 0.00	0.03 ± 0.00

Values are expressed as mean ± std.

**Table 7:** Calculated concentrations of total phenolic compounds and individual phenolics of crude skin extract in the bacterial suspensions.

Total and Individual Phenolics	Concentration of Crude Skin Extract in Antibacterial Test (mg/mL)			
	5.02	2.54	1.28	0.64
Total phenolic content (mg GAE/mL)	0.93 ± 0.06	0.47 ± 0.03	0.24 ± 0.01	0.12 ± 0.01
Malvidin-3-*O*-glucoside	63.40 ± 0.29	32.08 ± 0.15	16.16 ± 0.07	8.08 ± 0.04
Delphinidin-3-*O*-glucoside	3.90 ± 0.02	1.97 ± 0.01	0.99 ± 0.01	0.50 ± 0.00
Cyanidin-3-*O*-glucoside	1.38 ± 0.01	0.70 ± 0.01	0.35 ± 0.00	0.18 ± 0.00
Petunidind-3-*O*-glucoside	4.95 ± 0.04	2.51 ± 0.02	1.26 ± 0.01	0.63 ± 0.01
Peonidin-3-*O*-glucoside	1.93 ± 0.04	0.98 ± 0.02	0.49 ± 0.01	0.25 ± 0.00

Values are expressed as mean ± std.

## Data Availability

The unpublished data which may provide additional information in order to understand the present research are accessible from the corresponding author.

## Supplementary Materials

The following supporting information can be downloaded at: www.mdpi.com/article/10.3390/antibiotics14030236/s1, Figure S1: Myricetin-3-*O*-glucoside levels in grape skin and juice throughout the fermentation period. Figure S2: Quercetin-3-*O*-glucuronide in grape skin and juice throughout the fermentation period. Figure S3: Level of different anthocyanins in grape skin throughout the fermentation period. Figure S4: Level of different anthocyanins in grape juice throughout the fermentation period. Table S1: Calibration curve data, limit of detection (LOD), and limit of quantification (LOQ) for the HPLC analysis of phenolic compounds.
