# Supplementary material for: Insight into the Phenolic Composition of Cabernet Sauvignon Grapevine Berries During Fermentation—Towards the Application of Winery By-Products for Antibacterial Purposes
Source: Antibiotics (Basel). 2025 Feb 25;14(3):236. doi: 10.3390/antibiotics14030236 (PMC11939261; doi:10.3390/antibiotics14030236)
Supplement: Supplementary file 1 [file antibiotics-14-00236-s001.zip › antibiotics-3473496-supplementary.pdf]

antibiotics

MDPI

*Supplementary Materials*

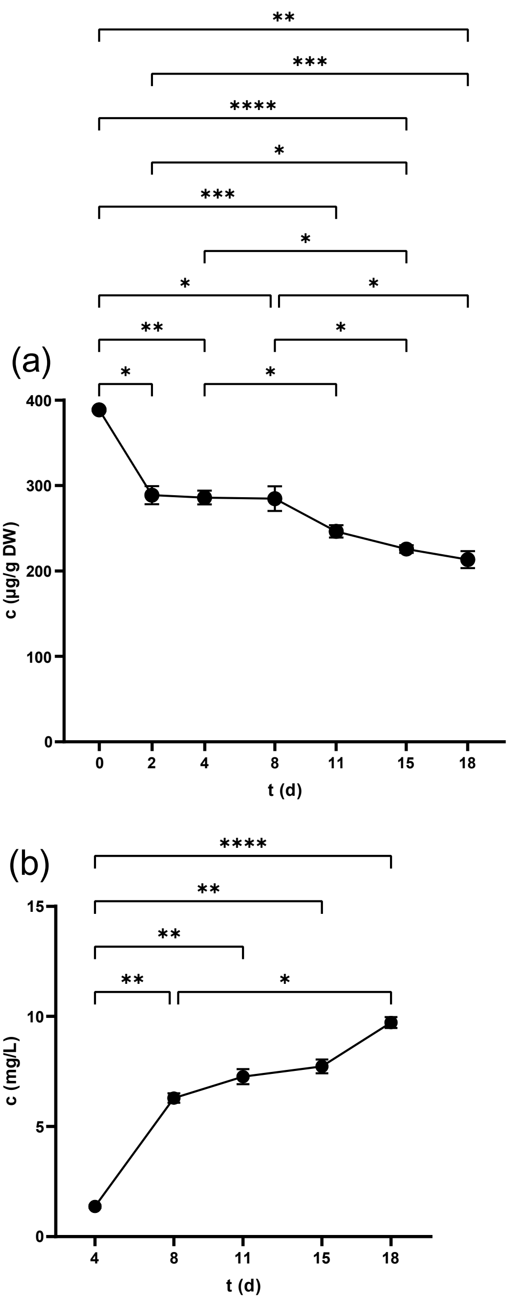


**Figure S1.** Myricetin-3-*O*-glucoside concentration in (a) grape skin and (b) grape juice throughout the fermentation period: *$p < 0.05$, **$p < 0.01$, ***$p < 0.001$, ****$p < 0.0001$.

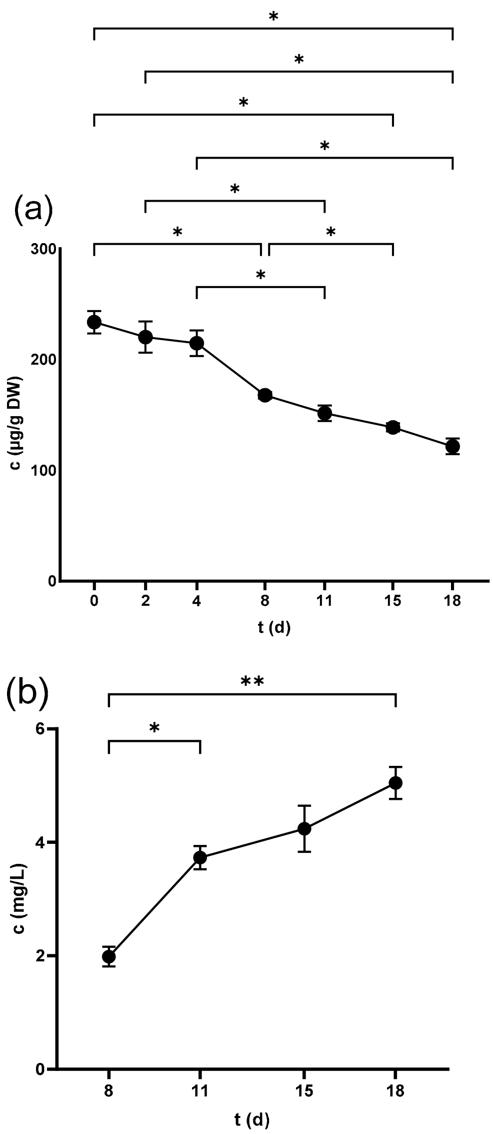


**Figure S2.** Quercetin-3-*O*-glucuronide concentration in (a) grape skin and (b) grape juice throughout the fermentation period: *$p < 0.05$, **$p < 0.01$, ***$p < 0.001$, ****$p < 0.0001$.

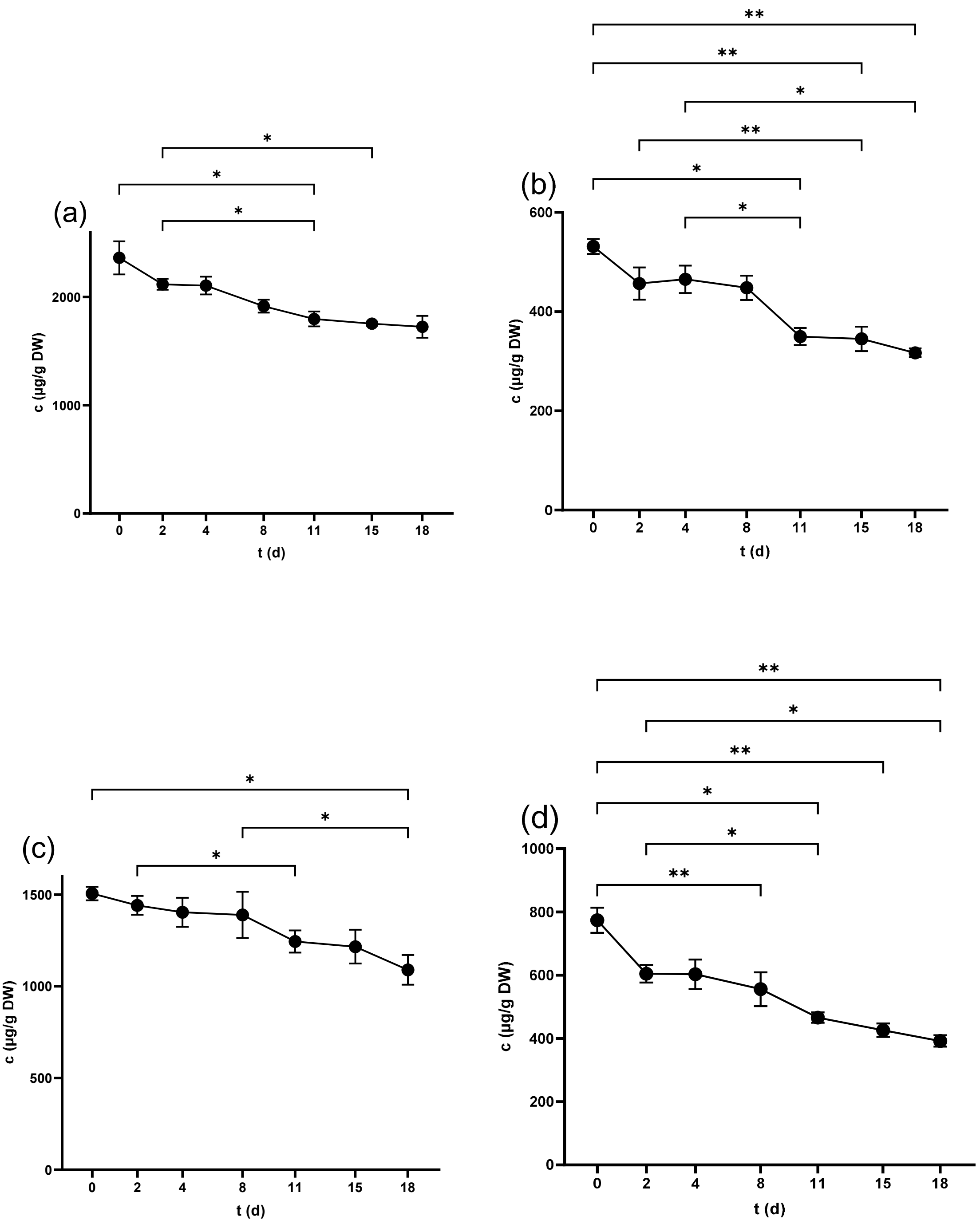


**Figure S3.** Anthocyanins: (a) Delphinidin-3-*O*-glucoside, (b) Cyanidin-3-*O*-glucoside, (c) Petunidin-3-*O*-glucoside, and (d) Peonidin-3-*O*-glucoside concentration in grape skins throughout the fermentation period.

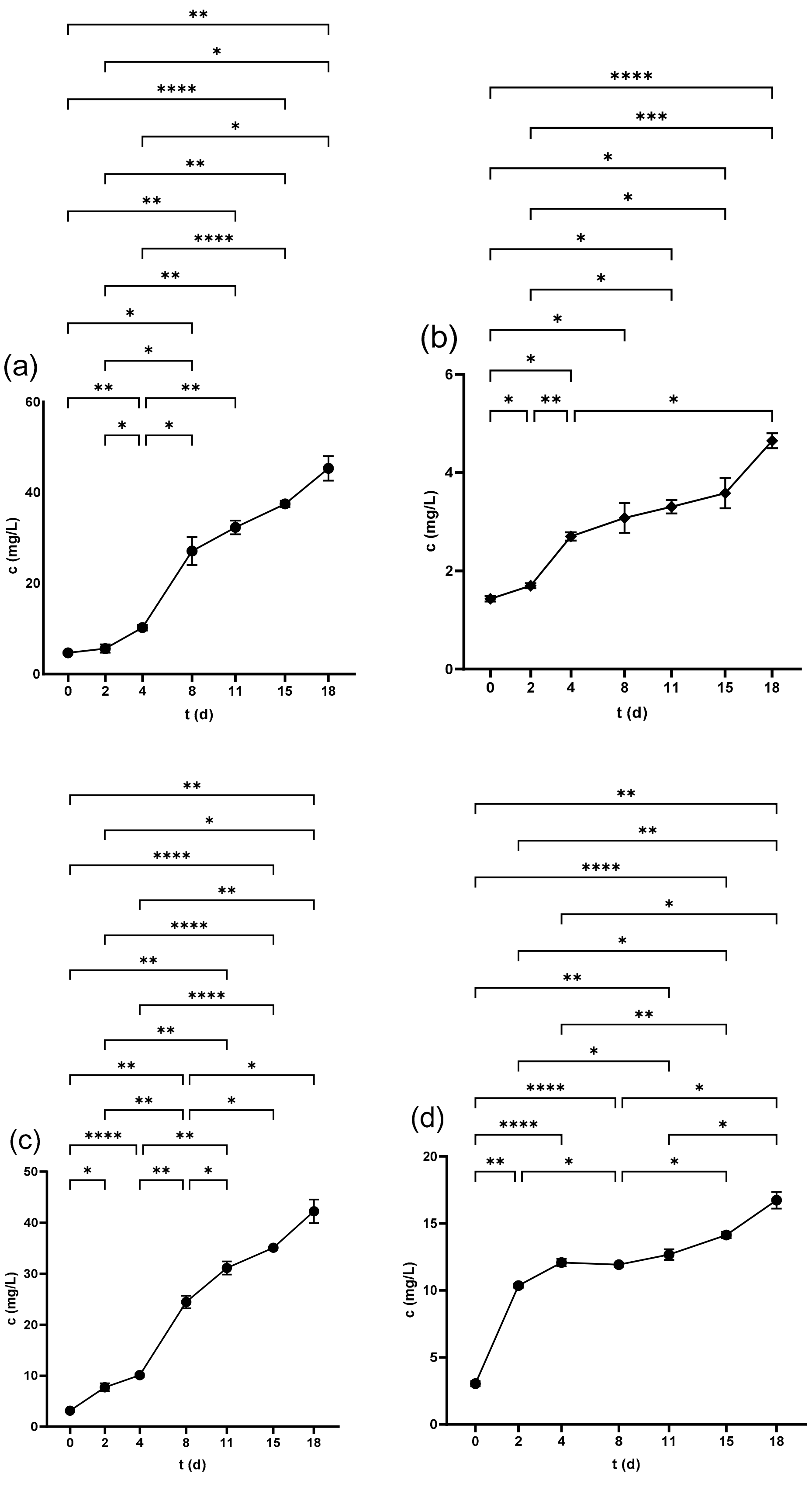


**Figure S4.** Anthocyanins: (a) Delphinidin-3-*O*-glucoside, (b) Cyanidin-3-*O*-glucoside, (c) Petunidin-3-*O*-glucoside, and (d) Peonidin-3-*O*-glucoside concentration in grape juice throughout the fermentation period: *$p < 0.05$, **$p < 0.01$, ***$p < 0.001$, ****$p < 0.0001$.

**Table S1.** Concentration ranges of the calibration, equations of calibration curves, correlation coefficients ($R^2$), limit of detection (LOD), and limit of quantification (LOQ) for HPLC analysis of phenolic compounds. Five concentration levels were used for the calibration of each analyte.

| Phenolic group | No. | Phenolic compound | Range of calibration (μg/mL) | Equation | $R^2$ | LOD (μg/mL) | LOQ (μg/mL) |
|---|---|---|---|---|---|---|---|
| flavan-3-ols | | | | | | | |
| | 1 | Catechin | 1.6 - 96 | y = 137408x - 36283 | 0.999993 | 0.01 | 0.04 |
| | 2 | Epicatechin | 1.8 - 108 | y = 123975x + 20806 | 0.999994 | 0.04 | 0.12 |
| flavonols | 1 | Myricetin-3-*O*-galactoside | 0.1 - 1.0 | y = 42 406.0x - 425.4 | 0.999986 | 0.01 | 0.02 |
| | 2 | Myricetin-3-*O*-glucoside | 0.1 - 1.6 | y = 40 387.0x - 1 051.9 | 0.999963 | 0.01 | 0.04 |
| | 3 | Quercetin-3-*O*-rutinoside | 0.1 - 1.9 | y = 34 159.2x + 23.6 | 0.999904 | 0.03 | 0.08 |
| | 4 | Quercetin-3-*O*-galactoside | 0.1 - 2.2 | y = 39 702.6x - 276.7 | 0.999854 | 0.04 | 0.11 |
| | 5 | Quercetin-3-*O*-glucoside | 0.1 - 1.5 | y = 49 274.1x - 789.5 | 0.999971 | 0.01 | 0.03 |
| | 6 | Quercetin-3-*O*-glucuronide | 0.1 - 1.2 | y = 35 081.9x - 457.0 | 0.999717 | 0.03 | 0.08 |
| | 7 | Kaempferol-3-*O*-rutinoside | 0.1 - 1.9 | y = 34 473.2x - 826.0 | 0.999894 | 0.03 | 0.08 |
| | 8 | Kaempferol-3-*O*-glucoside | 0.1 - 1.7 | y = 53 485.4x - 433.9 | 0.999970 | 0.01 | 0.04 |
| | 9 | Isorhamnetin-3-*O*-glucoside | 0.1 - 1.0 | y = 47 664.5x - 40.8 | 0.999758 | 0.02 | 0.06 |
| | 10 | Kaempferol-3-*O*-glucuronide | 0.1 - 1.0 | y = 46 398.2x + 544.5 | 0.999384 | 0.02 | 0.05 |
| anthocyanins | | | | | | | |
| | 1 | Delphinidin-3-*O*-glucoside | 1.6 - 32 | y = 15 646.3x - 6 445.6 | 0.999925 | 0.1 | 0.4 |
| | 2 | Cyanidin-3-*O*-glucoside | 0.9 - 18 | y = 14 471.2x - 4 468.1 | 0.999957 | 0.3 | 0.6 |
| | 3 | Pelargonidin-3-*O*-glucoside | 1.3 - 13 | y = 7 094.1x - 519.9 | 0.999992 | 0.1 | 0.2 |
| | 4 | Petunidin-3-*O*-glucoside | 1.1 - 22 | y = 14 096.4x - 4 398.6 | 0.999909 | 0.1 | 0.3 |
| | 5 | Peonidin-3-*O*-glucoside | 1.1 - 22 | y = 12 987.8x - 4 910.0 | 0.999964 | 0.2 | 0.5 |
| | 6 | Malvidin-3-*O*-glucoside | 1.0 - 20 | y = 13 509.1x - 3 564.8 | 0.999973 | 0.1 | 0.4 |
| stilbenes | 1 | Piceid | 0.1 - 5.3 | y = 20 167.4x - 305.0 | 0.999895 | 0.07 | 0.20 |
| | 2 | Resveratrol | 0.1 - 7.0 | y = 29 882.8x - 676.7 | 0.999918 | 0.08 | 0.25 |
